# Targeted and Untargeted Mass Spectrometry-Based Metabolomics for Chemical Profiling of Three Coffee Species

**DOI:** 10.3390/molecules27103152

**Published:** 2022-05-14

**Authors:** Andrea Montis, Florence Souard, Cédric Delporte, Piet Stoffelen, Caroline Stévigny, Pierre Van Antwerpen

**Affiliations:** 1 RD3 Unit of Pharmacognosy, Bioanalysis and Drug Discovery, Faculty of Pharmacy, Campus Plaine, Université Libre de Bruxelles, 1050 Brussels, Belgium; cedric.delporte@ulb.be (C.D.); caroline.stevigny@ulb.be (C.S.); pierre.van.antwerpen@ulb.be (P.V.A.); 2 APFP, Faculty of Pharmacy, Campus Plaine, Université Libre de Bruxelles, 1050 Brussels, Belgium; 3 Département de Pharmacochimie Moléculaire, UMR 5063 CNRS, Université Grenoble Alpes, 38400 Saint-Martin d’Hères, France; florence.souard@ulb.be; 4 Unit of Pharmacology, Pharmacotherapy and Pharmaceutical Care, DPP Department, Faculty of Pharmacy, Campus Plaine, Université Libre de Bruxelles, 1050 Brussels, Belgium; 5 Meise Botanic Garden, 1860 Meise, Belgium; piet.stoffelen@plantentuinmeise.be

**Keywords:** coffee leaves, *Coffea anthonyi*, food supplements, LC-(HR)MS, molecular networks

## Abstract

While coffee beans have been studied for many years, researchers are showing a growing interest in coffee leaves and by-products, but little information is currently available on coffee species other than *Coffea arabica* and *Coffea canephora*. The aim of this work was to perform a targeted and untargeted metabolomics study on *Coffea arabica*, *Coffea canephora* and *Coffea anthonyi*. The application of the recent high-resolution mass spectrometry-based metabolomics tools allowed us to gain a clear overview of the main differences among the coffee species. The results showed that the leaves and fruits of *Coffea anthonyi* had a different metabolite profile when compared to the two other species. In *Coffea anthonyi*, caffeine levels were found in lower concentrations while caffeoylquinic acid and mangiferin-related compounds were found in higher concentrations. A large number of specialized metabolites can be found in *Coffea anthonyi* tissues, making this species a valid candidate for innovative healthcare products made with coffee extracts.

## 1. Introduction

The intake of dietary supplements has widely increased in the last 20 years [1]. The European Food Safety Authority (EFSA) defines dietary supplements as products that contain ingredients such as vitamins, minerals, amino acids, essential fatty acids, fiber and herbal extracts. These supplements are intended to correct nutritional deficiencies, but they cannot be considered medicinal products. A large variety of these products have appeared on the market and many in vitro and in vivo studies have shown the efficiency of their components to prevent a large number of diseases such as colitis [2], neurological diseases [3], anxiety disorders and insomnia [4] or to improve the natural functionalities of the organism such as those of the immune system [5]. Coffee beans are currently used to make herbal remedies due to their high polyphenol content. Chlorogenic acids represent a large portion of the polyphenolic fraction of coffee tissues. This family of compounds includes caffeoylquinic acids (CQAs), feruloylquinic acids (FQAs) and coumaroylquinic acids (3-CoQA) in monomeric or dimeric form. These compounds could have a role in the attenuation of obesity and diabetes, or in the prevention of some chronic pathologies such as cancer [6,7]. Products with high content of chlorogenic acids from green coffee beans have been successfully marketed [8]. As beans, leaves could also be recommended for their high content of polyphenolic compounds [9]. In addition, the leaves of some coffee species provide a discrete content of xanthones, which have not always been detected in coffee beans. Mangiferin, the most well-known xanthone, is reported as a compound useful in the prevention of myocardial infarction, colitis, asthma or Parkinson’s disease [10]. Some countries promote the health properties of coffee leaves and use sun-dried coffee leaves as a tea substitute [11]. The EFSA approved the trade in coffee leaves of *C. arabica* and *C. canephora*, which could be marketed in the EU as a herbal infusion or as an ingredient for other beverages [12].

However, most of the available studies made on *Coffea* were carried out on *C. arabica* and *C. canephora*, and did not explore the qualities of the less studied coffee species. This research aims to explore and compare the metabolome of leaf and fruit aqueous extracts of a poorly studied species, *Coffea anthonyi*. Its metabolome will be compared with those of *C. arabica* and *C. canephora*. An overview of the chemical composition of the phloem sap collected from these species will also be given. *Coffea anthonyi* is endemic in Southeast Cameroon and the Northwest Republic of the Congo. It is a diploid species and shows morphological similarities with *C. eugenioides*, the maternal progenitor species of *C. arabica* [13,14]. Both species are separated from each other by a disjunction in the Congo Basin and their current habitat is different. *C. anthonyi* grows in low altitude forests (360–650 m) in Cameroon and the Republic of the Congo, while *C. eugenioides* is present in the mountain and sub mountain forests between 1000 and 2000 m in the Eastern parts of the Democratic Republic of Congo, in Uganda, Kenya, Rwanda, Burundi and southern Soudan. However, it is occasionally found at low altitudes as well [15]. *C. arabica*, which occupies first place in the coffee market, also grows better at high altitudes (more than 1000 m) [16]. The rapid climate changes have a bigger impact on species that grow at high altitudes. An increase of about two degrees Fahrenheit in the Earth’s average temperature was recorded during the 20th century [17], and the expansion of the “greenhouse effect” is considered the main cause of the current global warming trend [18]. Considering the current climatic conditions, it is possible that a large portion of the high-altitude forests including endemic coffee plants will gradually disappear and mountain crops’ viability (e.g., *C. arabica*) will be impacted. Consequently, *C. anthonyi* could be a good “low-land” alternative to the “highland” *C. arabica,* which is more threatened by global warming, for making coffee beverages or food supplements.

## 2. Results and Discussion

### 2.1. Coffee Leaf Metabolomics—ESI-MS (+) Mode

**Figure 1:**
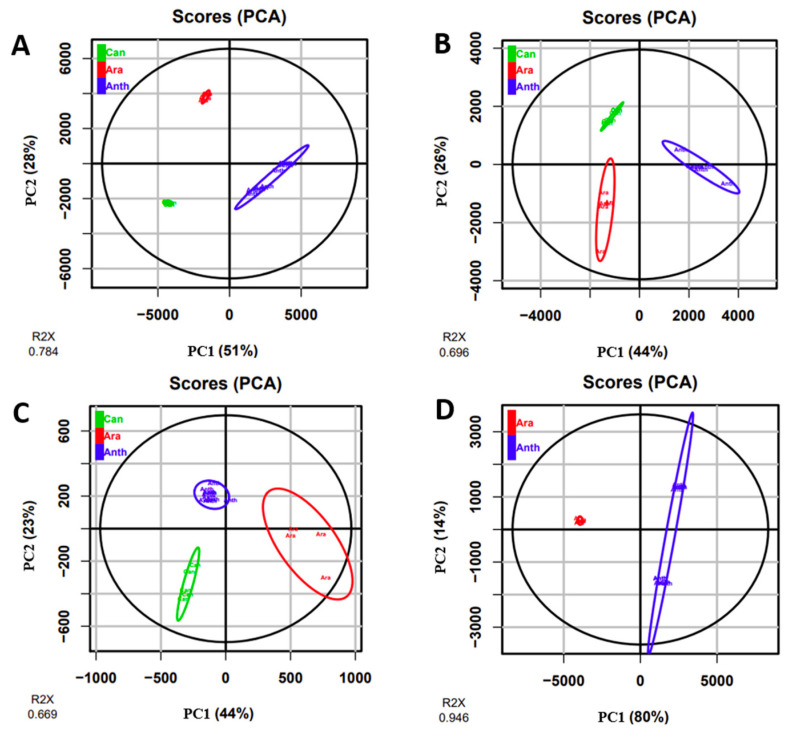
Principal component analysis (PCA) score plots showing the inter-species separation for the leaves analyzed in ESI-MS (+) mode (**A**), the leaves in ESI-MS (−) mode (**B**), the phloem sap in ESI-MS (+) (**C**), and the fruits in ESI-MS (+) (**D**). For all plots principal components 1 (PC1) and 2 (PC2) are presented. R²X scores are shown.

**Figure 2:**
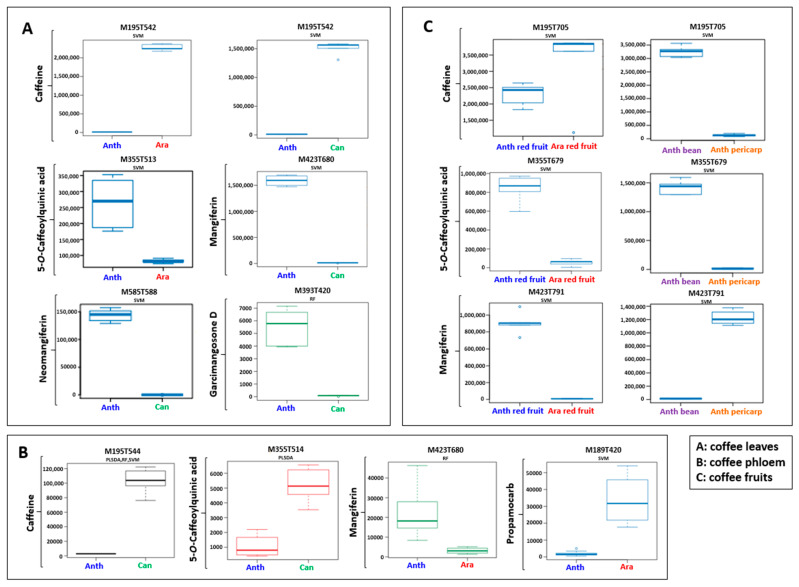
Biosigner boxplots showing the main features detected in the leaves (**A**), in the phloem sap (**B**) and in the fruits (**C**) of the three coffee species (Anth = *C. anthonyi*; Ara = *C. arabica*; Can = *C. canephora)*. The x-axis of the boxplots shows the coffee samples, while the y-axis shows the normalized intensities of the metabolites.

After analytical analysis, statistical multivariate analysis was carried out with principal component analysis (PCA) to first visualize the rough distribution of the groups according to two principal components (Figure 1A). The PCA score plot showed good inter-species discrimination with principal component 1 (PC1) and principal component 2 (PC2), explaining 51% and 28% of the total variation, respectively (Figure 1A). Loading plots show the main discriminant metabolites found in each cluster (see Supplementary Materials Figure S1A). In a recent study in our research group by Souard et al. [19], the three studied coffee species—*C. anthonyi*, *C. arabica* and *C. canephora*—were well clustered together, showing that the metabolite profiles of these three species were very close to each other when compared to the metabolome of the other seven coffee species which were analyzed by the authors. In contrast, our PCA analysis was able to discriminate the three species from each other illustrating that the metabolome was actually different. Indeed, the Biosigner algorithm [20] was used to highlight the features which were found to be the most discriminant for each sample (biomarkers of each species). SVM and RF algorithms selected the most statistically significant features (Figure 2A). The main markers returned by Biosigner were identified as caffeine, 5-CQA, mangiferin, neomangiferin and garcimangosone D. The boxplots clearly showed the distribution of the identified compounds in the leaves of the three coffee species. For instance, caffeine was detected in the leaves of *C. arabica* and *C. canephora* while *C. anthonyi* had a lower amount. However, the 5-CQA and the three mangiferin derivatives were more abundant in the *C. anthonyi* leaves. To confirm the results observed with Biosigner, a quantitative assay was carried out and the results are summarized in Table 1. The highest content of caffeine was found in the *C. arabica* leaves while the highest content of xanthone and CQA derivatives was found in the leaves of *C. anthonyi* (Table 1).

Therefore, the four families of compounds (purine alkaloids, chlorogenic acids, xanthones and benzophenones) (Supplementary Materials Table S1) have drawn our attention and were carefully investigated. They were first identified based on their exact mass, RT and according to the literature. Among the purine alkaloids, caffeine was already assigned to the ion with m/z 195.0874. Several CQA derivatives could be also identified based on their m/z and RT. First, the m/z 355.1025 ion was attributed to the main CQA isomer present in coffee tissues: 5-CQA. Two other ions m/z 355.1022 and 355.1020 with different RTs were identified and attributed to 4-CQA and 3-CQA isomers, respectively. The m/z 337.0918 ion with the putative formula C_16_H_17_O_8_ was defined as caffeoylshikimic acid while the m/z 517.1549 ion with the putative formula C_22_H_29_O_14_ was defined as a glucopyranosyl-CQA. An overview of the literature allowed us to attribute the m/z 369.0602 to a caffeoylquinic acid methyl ester, since FQAs were not previously detected in *C. anthonyi* leaves [21]. Among xanthones, several mangiferin derivatives were found. The m/z 423.0921 and 585.1451 were already assigned to mangiferin and neomangiferin, respectively, whereas compounds with m/z 437.1078, corresponding to the putative formulas C_20_H_21_O_11_ were assigned to homomangiferin. Moreover, a xanthone derivative with a dimeric structure was found. Indeed, the proposed formula C_38_H_35_O_22_ for the feature with m/z 843.1609 corresponds to a tetrahydroxyxanthone-*C*-hexoside dimer [22]. Some benzophenone derivatives were also investigated. The ions with m/z 393.1182 with the formulas C_19_H_21_O_9_ were observed in Biosigner and putatively assigned to a 2-benzoyl-3,5-dihydroxyphenyl beta-D-glucopyranoside, also called garcimangosone D. Another ion has drawn our attention, m/z 571.1655, with the formula C_25_H_31_O_15,_ which might be assigned to an iriflophenone-di-*O,C*-hexoside isomer [22].

**Figure 3:**
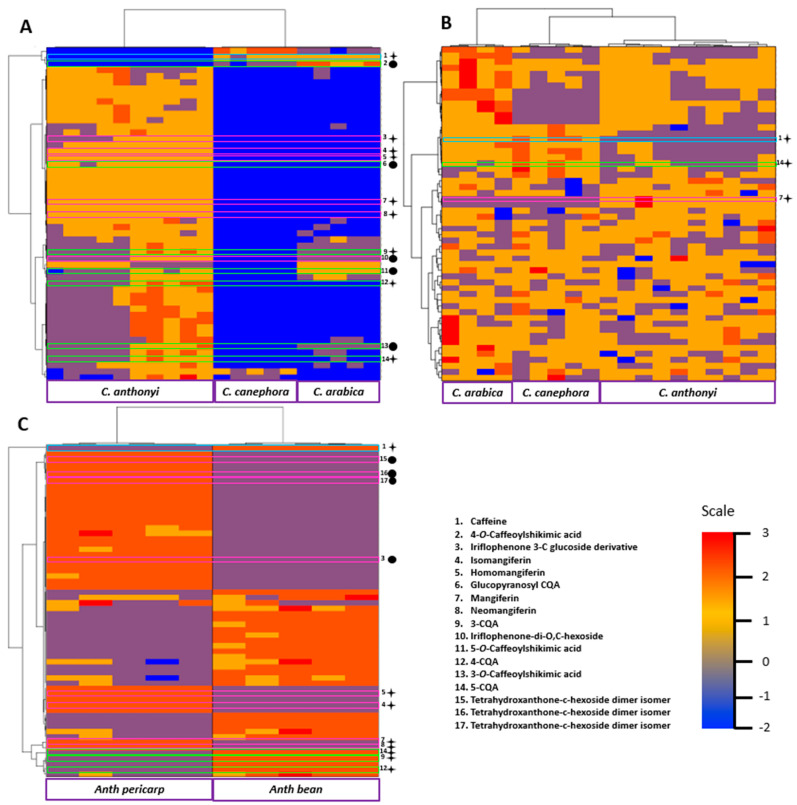
Heatmaps showing the distribution of most discriminant metabolites detected in ESI-MS (+) mode in the leaves (**A**), in the phloem sap (**B**) and in the fruits (**C**) of the analyzed species. ● = annotation based on the literature. ✦ = annotation based on molecular networks and/or by the injected standard compound. Light blue boxes = caffeine; green boxes = CGAs; pink boxes = xanthones and benzophenones. The below x-axis represents the coffee species or fruit part while the above x-axis shows the dendrogram that discriminates samples from each other according to the sample type. The y-axis represents the dendrogram that separates the samples according to the presence of metabolites.

In Figure 3, the heatmap represents the relative abundance of the features detected (raw) in each sample (column) [23] that was classified in order to illustrate the similarities between samples (Figure 3A). The dendrogram designed on the y-axis pointed out that the first separation between metabolites (upper blue lines) already demonstrated separation between *C. anthonyi* on one side and *C. arabica* and *C. canephora* on the other side. This could be explained by the fact that the metabolome of *C. arabica* leaves is similar to that of *C. canephora* leaves. Among these metabolites, caffeine was present in a lower amount in *C. anthonyi* as previously illustrated by Biosigner. The second separation allowed the discrimination of two families of samples in *C. anthonyi* while the third illustrated the lowest amount of CQAs found in *C. canephora* leaves compared to the two other species. This could be explained by the fact the CQA derivatives are synthesized in the youngest leaves of *C. canephora* and are then transferred into the phloem sap when the leaf is adult [24]. Finally, the levels of benzophenone derivatives were also found higher in *C. anthonyi* leaves. These results can be explained by the fact that benzophenones are the main precursors of xanthones. Indeed, the only difference between their basic structure is the oxygenated heterocycle of xanthones, which is not yet totally closed in the structure of benzophenones. A slight difference in the levels of some metabolites, such as CGAs, is detectable in the two plants of *C. anthonyi* used to build the heatmap. This could explain why both yellow and violet colors in the lowest part of the heatmap correspond to *C. anthonyi* samples.

### 2.2. Coffee Leaf Metabolomics—ESI-MS (−) Mode

Three different species of *C. anthonyi*, *C. arabica* and *C. canephora* were also analyzed in ESI-MS (−) mode and the dataset included more than 4000 features. The PCA was performed and the score plot showed three clusters that were well separated. PC1 and PC2 explained 44% and 26%, respectively, of the total variation (Figure 1B). Loading plots show the main discriminant metabolites found in each cluster (see Supplementary Materials Figure S1B). A detailed annotation of the metabolites previously investigated was carried out (see Supplementary Materials Table S1). The three isomers of CQAs provided m/z 353.0890, m/z 353.0888 and m/z 353.0887 as [M − H]^−^ ions as expected and the three isomers of FQAs could also be identified (m/z included between 367.1038 and 367.1044). Mangiferin occurred at m/z 421.0802. The performed ESI-MS (−) approach allowed the identification of isomangiferin with a similar m/z value to that of mangiferin but with a slight difference in the retention time. The presence of the other mangiferin derivatives such as homomangiferin and neomangiferin could be confirmed by the m/z 435.0946 and 583.1322 corresponding to the putative formulas C_20_H_19_O_11_ and C_25_H_28_O_16_, respectively. The dimeric xanthone derivative with m/z 841.1480 and both benzophenone derivatives, garcimangosone D with m/z 391.1048, and iriflophenone-di-*O,C*-hexoside with m/z 569.1521 were also identified. Interestingly, the content of CQAs and xanthone derivatives was found high in *C. anthonyi* leaves but the levels of FQAs were found low when compared to the level of these compounds in the leaves of the two other species.

### 2.3. Coffee Phloem Metabolomics—ESI-MS (+) Mode

Since the phloem sap is considered an important highway for the translocation of some compounds [25], it was extracted from the three coffee species and a metabolomics analysis was carried out. A total of 57 features were detected. Unsupervised multivariate analyses were performed on the phloem of the three coffee species. Despite the increase in intra-species variability, the PCA plot clearly showed three different clusters, according to the three analyzed species. PC1 and PC2 captured 44 % and 23 %, respectively, of the total variation (Figure 1C). Loading plots show the main discriminant metabolites found in each cluster (see Supplementary Materials Figure S1C). The intra-species variability could be explained by the difficulty of the sample preparation which provides random errors (see Materials and Methods (Section 3.3)). Among the metabolites the main discriminant ones returned by Biosigner were again caffeine with m/z 195.0886, 5-CQA with m/z 355.1026 and mangiferin with m/z 423.0921, showing that the phloem sap could be representative of the metabolites in the leaves. However, discrepancies must be observed. First, the three algorithms (PLS-DA, RF and SVM) of the tool concurred to select caffeine as a marker of *C. canephora* phloem sap (Figure 2B). More precisely, the content of caffeine in *C. canephora* phloem sap was 0.8 ± 0.2 mg/L while its content in *C. arabica* phloem sap was 0.04 ± 0.01 mg/L. Caffeine was not detected in the phloem of *C. anthonyi*. These results were different from the leaves as the caffeine content was higher in *C. arabica* (Figure 2A). Regarding the CQA derivatives only, the ion [M + H]^+^ with m/z 355.1026 assigned to the 5-CQA isomer was detectable in low amounts. Despite the high amount of 5-CQA in the *C. anthonyi* leaves, the levels of 5-CQA were found higher in *C. canephora* phloem sap. The similar repartition among caffeine and CQAs in the phloem of this species could be explained by the complexation which can occur between these metabolites [26]. In *C. arabica,* the content of 5-CQA in the phloem was found to be lower, and according to “complexation theory” the content of caffeine was also found lower. In contrast, mangiferin was found as a marker of *C. anthonyi* phloem sap which was in accordance with the results in the leaves (Figure 2A). A fourth compound with m/z 189.1592 was also returned by the Biosigner (Figure 2B). This compound was identified as a pesticide. Particularly, its calculated formula C_9_H_21_N_2_O_2_ corresponded to that of propamocarb, which is often nebulized on coffee plants to prevent the fungal infections in the Meise Botanic Garden, as confirmed by the gardeners.

The heatmap shows that the phloem sap of the three coffee species had different chemical compositions despite the difficulties encountered during the extraction process (Figure 3B). Indeed, premature healing of petioles or the accidental extraction from the leaf surface during the process could explain why the detected metabolites were found in very low concentrations in the extracts. Interestingly, the heatmap first separated *C. anthonyi* and *C. canephora* from *C. arabica* based on the differences detectable in the highest part of the heatmap. Then the two first species were separated based on the different content of caffeine, CQAs and mangiferin-related compounds. This same clustering was also observable in the PCA. Indeed, the PC1 component clearly indicated a first remarkable separation of *C. anthonyi* and *C. canephora* phloem sap from *C. arabica* phloem sap.

As shown by the results, caffeine, mangiferin and CGAs can be transferred from the leaves to the phloem sap, and the content of these metabolites can vary depending on the species. Some xanthone compounds such as mangiferin can be in the phloem sap of the species which synthesizes these molecules in the leaves, while they are undetectable in coffee species that produce fewer amounts of xanthones in the leaves. Interestingly, CGA distribution in the phloem sap is strictly related to the species and the content of CGAs in the sap does not depend on the levels of these compounds in the leaves. Indeed, it was observed that species with a high content of CGAs in the leaves such as *C. anthonyi*, do not show a related high content of these metabolites in the phloem sap. In contrast, in *C. canephora*, a higher content of CGAs was detected in the phloem sap, while low levels of these compounds were detected in the leaves. Caffeine levels in the phloem sap seem to be related to the levels of CGAs.

### 2.4. Coffee Fruit Metabolomics—ESI-MS (+) Mode

*C. arabica* fruits have been explored for a long time, with this species being the main employed coffee species used to make the coffee beverage. However, little is known about *C. anthonyi* fruits. Extracts were prepared and more than 3000 features could be observed. Green fruits and red fruits of *C. anthonyi* were analyzed. In addition, beans and pericarps of red fruits were also analyzed separately and their metabolomes were compared to that of *C. arabica* red fruits. Under the culture conditions at the Meise Botanic Garden, unfortunately, *C. canephora* did not produce fruits as it is self-incompatible and pollinators were not present during our time study. PCA displayed *C. anthonyi* fruits and *C. arabica* fruits as two separated groups (Figure 1D). Loading plots show the main discriminant metabolites found in each cluster (see Supplementary Materials Figure S1A). The majority of metabolites found in the leaves of *C. anthonyi* could also be found in the fruits of the same species. The peak of protonated ion [M + H]^+^ corresponding to the caffeine was present at m/z 195.0870, meaning that *C. anthonyi* produces caffeine in beans at a higher level than in the leaves. Three peaks with m/z 355.1015, 355.1018 and 355.1015 corresponding to the three 5-, 4- and 3-CQA isomers were identified and their identity was confirmed by observing the same three different retention times of the injected standard samples. Moreover, a peak corresponding to caffeoylshikimic acid at m/z 337.0921 was also present. Mangiferin, homomangiferin and neomangiferin occurred at m/z 423.0924, 437.1073 and 585.1453. The same dimer with m/z 843.1611 found in the leaves could also be identified in the fruits. Biosigner revealed that also, in this case, caffeine was the key metabolite to discriminate *C. anthonyi* from *C. arabica* fruits. The boxplots show that the highest levels of caffeine were detected in *C. arabica* fruits (Figure 2C). In *C. anthonyi,* this xanthine accumulates more in the beans than in the leaves. Regarding the CQA derivatives, 5-CQA was mainly present in the fruits of *C. anthonyi*. More specifically, CQAs were fully stocked in the beans and almost absent in the pericarp (Table 2). The histolocalization of these compounds strengthens the hypothesis of the formation of a complex between caffeine and CQAs as they were again co-localized in the beans. A similar distribution of these compounds was found in *C. arabica* fruits. According to Koshiro et al., caffeine and chlorogenic acids are almost fully stocked in the beans of this species and not in the fruit pericarp [27,28]. Mangiferin was found in a higher amount in *C. anthonyi* fruits. However, the levels of all the xanthone derivatives were found to be higher in the pericarp, being almost absent in the beans. Mangiferin was not detected in *C. arabica* fruits (Table 2). In a previous study, a similar histolocalization of mangiferin was found in the fruits of *C. arabica* ‘Laurina’ and *C. pseudozanguebariae*. In both species, mangiferin was found in the fruit pericarp but not in the seeds. However, this xanthonoid was almost undetectable in *C. arabica* fruits [21]. The heatmap gives a clear view of the distribution of all focused metabolites in both the pericarp and beans of *C. anthonyi* fruits. (Figure 3C). Due to a large number of features, the variable metadata was filtered based on the m/z values and retention times before building the heatmap. This step allowed us to obtain a clear overview of the focused compounds. It can be concluded that mangiferin derivatives can be considered the main markers of *C. anthonyi* fruits as well as of *C. anthonyi* leaves. Until now, mangiferin and its derivatives were not largely described in coffee tissues. Some previous information was reported for *C. anthonyi* leaves by Campa et al. [21] regarding the content of mangiferin, but no details about the presence of global xanthones in its tissues were given. Some other information was given about the content of mangiferin in *C. salvatrix* leaves. Therefore, most of the information regarding the presence of these derivatives in *Coffea* concern the leaves and not the fruits.

### 2.5. Molecular Networks

To confirm the annotation of the compounds detected in positive mode and to investigate further metabolites in the three coffee species, a molecular network (MN) was carried out on the coffee leaves dataset by setting a cosine score of 0.5. Only the networks including at least two or more features linked to each other were analyzed.

**Figure 4:**
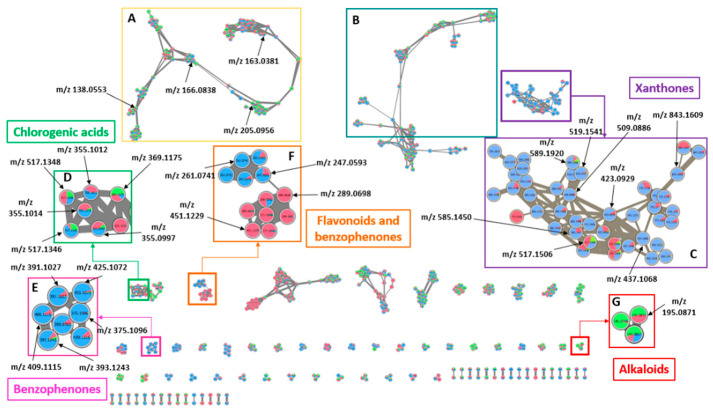
Molecular network of coffee leaf samples showing networks of aminoacids, trigonelline and caffeic acid derivatives (**A**), long-alkyl-chain compounds (**B**), xanthones (**C**), CGA derivatives (**D**), benzophenones (**E**) benzofurans and flavanone derivatives (**F**), and xanthine alkaloids (**G**). the colour in the circles represent the relative abundance in the species with blue: *C. anthonyi*, red: *C. arabica*, green: *C. canephora*.

Figure 4A shows a cluster including many m/z values corresponding to small molecules such as trigonelline. Trigonelline appears at m/z 138.0553 (RT = 2.39 min, δ = −2.52 ppm) and the annotation was performed by comparing the spectrum of the target compound with the spectrum of the standard compound implemented in MetGem libraries. The CS associated with the spectrum of the standard compound was about 0.61. Interestingly, this compound was mainly detected in the leaves of *C. canephora*. Some caffeic acid derivatives were also detected at m/z 163.0381 (RT = 8.39 min). The same cluster also included some amino acids, such as phenylalanine at m/z 166.0729 (RT = 4.24 min, δ = −14.87 ppm) and tryptophane at m/z 205.0956 (RT = 6.92 min, δ = 7.61 ppm). While phenylalanine was mainly detected in the leaves of *C. anthonyi* and *C. arabica*, tryptophane was found to be predominant in the leaves of *C. canephora*. Figure 4B shows a cluster with features corresponding to molecules with long alkyl chains.

Figure 4C shows one of the most interesting clusters of the network. Most of the features of this cluster were related to xanthone derivatives. The three nodes corresponding to mangiferin, homomangiferin and neomangiferin were identified. Particularly, the GNPS library could confirm the presence of mangiferin (m/z = 423.0929, RT = 10.0 min, δ = −1.69 ppm) by matching the experimental mass spectrum with the one in the library. The CS value was 0.89 with seven main matching fragments: m/z 273.0389, 303.0497, 327.0518, 339.0471, 369.0567, 299.0554, 351.0504. The fragment with m/z 303.0497 could be formed after the loss of a C_4_H_8_O_4_ unit from the chemical structure of mangiferin corresponding to the successive fragmentations of the oxane cycle (M-C_3_H_6_O_3_-CH_2_O), while the m/z 273.0389 (M- C_3_H_6_O_3_-CH_2_O-CH_2_O) can be progressively formed from the ion with m/z 303.0497 after the loss of a CH_2_O fragment [29]. The node with m/z 437.1068 (RT = 11.10 min; δ = 2.38 ppm) was linked to the node of mangiferin with a CS of 0.97, suggesting that this M + H^+^ ion could be attributed to homomangiferin; moreover, its m/z value was associated to that of the standard compound of the MetGem database with a CS = 0.74. The fragments with m/z = 317.0631 and 287.0525 corresponding to the fragments at m/z = 303.0497 and 273.0389, previously found for mangiferin, were detected. The only difference was the presence of a methoxy on C5 of the xanthone unit to the previous fragments, which is missing in the chemical structure of mangiferin conferring a delta mass of 14 m/z (+CH_2_). This cluster also revealed the presence of a feature with m/z 585.1450 (RT = 8.59 min) corresponding to the delta mass of m/z 162 (+C_6_H_10_O_5_, sugar). Moreover, it provided more than seven matching fragments with those of mangiferin, confirming the previous annotation by Biosigner of this compound as neomangiferin (δ = −0.02 ppm). The presence of the fragments with m/z = 303.0456 and 273.0290 were also detected in the spectrum of the feature with m/z = 509.0886. In addition, the high CS value (0.73) linking this feature with the node of mangiferin confirmed that this ion is a xanthone derivative. A node with an m/z of 843.1609 (RT = 7.14 min) was also found. The standard search algorithm available in MetGem has not proposed any matching compounds for this mass. However, the fact that this ion was found in the cluster including the xanthone derivatives suggested that it could correspond to the tetrahydroxyxanthone-*C*-hexoside dimer (δ = −0.65 ppm). Moreover, two fragments with m/z 723.1141 and 603.0724 were detected in the spectrum. A difference of 120 m/z units between these two features was found, corresponding to four CH_2_O units, which is the same fragment lost by mangiferin (2 CH_2_O). The node with m/z 517.1506 (RT = 7.75 min) mainly detected in *C. arabica* was related to the spectrum of an ion with m/z 355.1019 corresponding to a 5,7-dihydroxy-2-methyl-8-[(2S,3R,4R,5S,6R)-3,4,5-trihydroxy-6-(hydroxymethyl)oxan-2-yl]chromen-4-one (CS = 0.69, δ = 1.3 ppm) plus the delta mass of m/z 162 (+C_6_H_10_O_5_, hexose), which corresponds to the chromanone structure also found in xanthones but with a missing aromatic ring, which would contribute to providing the basic structure of xanthones. The spectrum of the features at m/z 509.0886 (RT = 12.61 min), 519.1541 (RT = 15.46 min) and 589.1920 (RT = 15.19 min), showed the two peaks with m/z 303.0497 and 273.0389, previously found in the spectrum of mangiferin, suggesting that these molecules are structurally related to mangiferin.

Figure 4D showed a cluster where the three CQA isomers were strongly related to each other, with an upper CS value of 0.90. The three features with m/z 355.0997 (RT = 5.70 min), 355.1014 (RT = 7.77 min), and 355.1012 (RT = 9.37 min) were attributed to 3-*O*-, 5-*O*- and 4-*O*-caffeoylquinic acid, respectively (δ = 7.51, 2.71 and 3.27 ppm). Moreover, a node with an m/z of 369.1175 (RT = 10.78 min) was identified and its spectrum matched that of feruloylquinic acid proposed by the MetGem database (CS = 0.96, δ = 1.38 ppm). Two other CQA derivatives related to the three main CQA isomers were found. Their m/z of 517.1346 (RT = 14.10 min) and 517.1348 (RT = 13.85 min) were attributed to two different diCQAs (dicaffeoylquinic acids) (δ = −1.06 and −1.45 ppm). The annotation was performed by comparing their spectrum to that of the standard compound. The fragment at m/z 163.0321 (M-C_16_H_17_O_8_) was found in both spectra of standard and target compounds, suggesting the loss of a C_16_H_17_O_8_ unit, which follows the breaking of the ester bond between caffeic acid and the second CQA molecule. Another matching fragment was found at m/z 164.0350, which can be formed after the loss of a C_7_H_11_O_6_ unit corresponding to the quinic acid structure. Moreover, a fragment at m/z = 319.0746 was also detected. This fragment can be formed after the loss of caffeic acid and two molecules of water (M-C_9_H_8_O_4_-2H_2_O). The presence of the fragment at m/z 164.0350 in both spectra of the CQA and diCQA isomers and the high CS value (0.92) confirmed that these molecules belong to the same family of polyphenolic derivatives. Moreover, as shown by Wu et al. [30] in a previous investigation of the fragmentation mechanism of caffeic acid derivatives, the fragment at m/z = 145.0290 formed after the loss of an H_2_O molecule from the fragment with m/z = 163.0321 was also found in all detected CQA derivatives.

Figure 4E showed a group of benzophenone derivatives. Particularly, a feature with m/z = 409.1115 (C_19_H_20_O_10_, RT = 7.52) was identified as a (4-hydroxyphenyl)-[2,4,6-trihydroxy-3-[2S,3R,4R,5S,6R)-3,4,5-trihydroxy-6-(hydroxymethyl)oxan-2-yl]phenyl]methanone or iriflophenone 3-C-glucoside (δ = 3.49 ppm) by comparing its spectrum with that of the standard compound (CS = 0.91). As shown by Berardini et al. [31], the first step of 3-C-glucoside benzophenones fragmentation is the partial loss of the oxane cycle (M-C_2_H_8_O_4_), which corresponded to the detected fragments at m/z = 313.0696 in our spectrum. Another important fragmentation is the loss of the phenol group on the benzophenone (Yu et al., 2013), which occurred concomitantly with the fragmentation of the oxane cycle. This provided fragments at 231.0276 m/z (M-C_6_H_5_O-CH_2_O-3 H_2_O), 219.0281 (M-C_6_H_5_O-C_2_H_8_O_4_-H), and 195.0251 (M-C_6_H_5_O-C_4_H_9_O_4_). Moreover, a fragment at m/z = 121.0280, corresponding to the formula C_7_H_5_O_2_, was detected in both spectra of standard and target compounds and was attributed to a hydroxyphenylmethanone. Another feature with m/z = 425.1072 (RT = 5.70) was directly linked to the iriflophenone C-3 glucoside and was identified as the maclurine 3-C-glucoside (C_19_H_20_O_11_) with one oxygen atom more (δ = 1.5 ppm). Indeed, a first fragment m/z = 329.0601 was observed and corresponded to the fragmentation of the oxane cycle (M-C_2_H_8_O_4_) where the oxygen atom is conserved on the dihydroxyphenyl cycle of the benzophenone. Moreover, fragments at 231.0276 m/z (M-C_6_H_5_O_2_-CH_2_O-3 H_2_O), 219.0281 (M-C_6_H_5_O_2_-C_2_H_8_O_4_-H), and 195.0251 (M-C_6_H_5_O_2_-C_4_H_9_O_4_) were also conserved while the fragment corresponding to the hydroxyphenylmethanone at m/z = 121.0280 disappeared. Two other features with m/z = 391.1027 (C_19_H_18_O_9_, RT = 7.38 min) and 393.1243 (RT = 6.38 min) directly linked to the previous compound (CS = 0.79 and 0.93) were found. The ion at m/z = 393.1243 could probably be the feature with m/z = 409.1115 less an oxygen atom in the trihydroxyphenyl of the benzophenone structure. This supposition was based on the presence of the fragment at m/z = 121.0280 (C_7_H_5_O_2_) that was found in the spectra of both compounds and the loss of the fragment at m/z = 195.0251 (C_19_H_20_O_10_-C_6_H_5_O-C_4_H_9_O_4_), which was replaced by a fragment at m/z = 179.0367 (C_19_H_20_O_9_-C_6_H_5_O-C_4_H_9_O_4_). This suggested that they belong to the same family of benzophenone derivatives but with a dihydroxylphenyl cycle. MN was useful in this case to properly identify this feature. Indeed, MN allowed us to annotate it as the iriflophenone 3-C-glucoside and not as garcimangosone D. Finally, it could be possible to detect a feature at m/z = 375.1096 (C_19_H_20_O_8_, RT 6.43 min.), corresponding to the feature at m/z = 391.1027 without the hydroxyl group on the phenol cycle of the benzophenone as suggested by the loss of the fragment at m/z = 121.0280 (C_7_H_5_O_2_). Moreover, in most of the spectra, the fragments at m/z 195.0280, 177.0172 and 165.0165 described by Beelders et al. [22] were detected.

Therefore, Figure 4F clearly shows two groups of features slightly related to each other and mainly found in *C. anthonyi* and *C. arabica* leaves. A fast investigation of libraries allowed us to assign to the ions mainly found in *C. arabica* leaves the structure of flavanone derivatives. The GNPS library matched the chemical structure of dihydrokaempferol with the m/z = 289.0698 (C_15_H_12_O_6_, δ = 1.5 ppm, RT = 9.92 min) feature with that of flavanone derivative in a [M + H]^+^ form, by comparing the target spectrum with that of a standard compound (CS = 0.94). The main matching fragment was found at m/z = 153.0176, which corresponds to the fragment C_7_H_4_O_4_ formed after the classical breaking of the ring C of flavanone [32]. Other fragments at m/z = 215.0703, 149.0171, and 107.0442 were also specific to this compound. The node corresponding to this mass was tightly linked to a node with m/z = 451.1229 (C_21_H_22_O_11_, RT = 9.40), which was attributed to a 3-O-glucoside dihydrokaempferol (δ = 1.31 ppm). Among the matching fragments, the m/z = 153.0126 was found after the neutral loss of the sugar (m/z = 289. 0695; C_21_H_22_O_11_-C_6_H_12_O_6_). Two features among the compounds mainly found in *C. anthonyi* leaves were related to a benzophenone structure according to the analog database. The ion with m/z 247.0593 (RT = 6.71 min) was associated with the structure of the iriflophenone (C_13_H_10_O_5_) non-glycosylated with the addition of a hydroxyl group (δ = 3.25 ppm). The CS value was about 0.64. Among the matching fragments, the signal at m/z = 153.0179 was found. This fragment could correspond to the fragment C_7_H_4_O_4,_ which can also be formed after the breaking of the flavanone cycle. The feature with m/z 261.0741 (RT = 13.16 min) was also related to the iriflophenone. This feature was related to the previous ion with m/z = 247.0593 by a CS = 0.95. Indeed, the only difference could be the addition of a (+CH_2_) group conferring a delta mass of +14 m/z. This difference was highlighted by the detection of a fragment at m/z 167.0279 instead of at m/z 153.0179. The fragment at 121.0228 was detected in the spectrum of both compounds, suggesting that the fragmentation involved the loss of the hydroxyphenylmethanone (C_7_H_5_O_2_). The presence of a slight edge (CS = 0.51) connecting the two subclusters could be explained by the fact that the non-glycosylated iriflophenone derivatives and flavanones have a common fragmentation pattern.

Figure 4G shows a node corresponding to the m/z of caffeine (C_8_H_10_N_4_O_2,_ m/z = 195.0871, RT = 8.36 min, δ = 2.84 ppm). Its spectrum was related to that of the standard compound (CS = 0.87). Among the matching fragments, the most intense one was detected at m/z = 138.0679. This fragment is formed after the breaking of the pyrimidine cycle and the loss of an M-CH_3_NCO unit, while the imidazole is not involved in the fragmentation process [33]. The color mapping confirmed the presence of this compound in the leaves of *C. arabica* and *C. canephora*.

While *C. arabica* and *C. canephora* have been studied for many years and their leaf and fruit chemical composition is almost fully known, *C. anthonyi* is only recently formally described as a new species [14] and as a consequence far less studied. The results of this study highlighted that *C. anthonyi* leaves are almost caffeine-free, while in its fruits caffeine could be detected but in lower amounts compared to *C. arabica* fruits. This means that this species could be employed to make products with a lower psychostimulant activity compared to the beverages made with *C. arabica*. Moreover, the high levels of mangiferin derivatives and CGAs in *C. anthonyi* could make it a useful coffee species for the prevention of several diseases, by exerting a cardioprotective, anti-inflammatory or anti-diabetic effect [10]. The metabolomics analysis also allowed us to detect the differences between the entire metabolome of the leaves and one of the fruits of the same species. It was interesting to note that the main remarkable difference was detected in caffeine levels. Caffeine was found at high levels in the fruits but it was undetectable in the leaves. The xanthone and CGA derivatives were found in high amounts in both leaves and fruits of *C. anthonyi*. In *C. arabica,* higher levels of mangiferin and CGAs were detected in the leaves when compared to the fruits, where mangiferin was not detected at all. The caffeine content was found to be high in the leaves as well as in the fruits of *C. arabica*. *C. canephora* leaves also showed a high amount of caffeine, while the other investigated polyphenolic compounds were almost undetectable. Consequently, if *C. anthonyi* will show good organoleptic properties, further development of beverages or other dietary supplements made with this coffee species could be envisaged. Moreover, as shown by the results, due to the high content of mangiferin derivatives and the absence of caffeine in the fruit pericarp of this species, *C. anthonyi* cherry pulp could also be exploited. As the coffee cherry pulp is already consumed in Ethiopia, Bolivia and Yemen, EFSA recently published a report where it was declared that they do not raise safety objections to the placing on the market of the cherry pulp from *C. arabica* and *C. canephora* [34].

## 3. Materials and Methods

### 3.1. Chemicals and Reagents

Ultra-high purity water was obtained by filtration using a Milli-Q system from Millipore (Bedford, MA, USA). LC-MS quality acetonitrile, formic acid (FA) and trifluoroacetic acid (TFA) were purchased from Fisher Scientific (Waltham, MA, USA), while caffeine (99%), 3-CQA (>99%), 4-CQA (>99%), 5-CQA (>99%) and mangiferin (>99%) were purchased from Sigma-Aldrich (Steinheim, Germany).

### 3.2. Coffee Samples

Seventy samples of leaves from 3 different coffee species (*C. arabica*, *C. anthonyi* and *C. canephora*) were collected in December 2018 in the tropical greenhouse of Meise Botanic Garden. Ten samples of fruits from *C. arabica* and *C. anthonyi* were also collected. Leaves and fruits (entire drupes) were harvested in the morning between 9 am and 12 am. All plants have grown in the tropical greenhouses of Meise Botanic Garden (Meise, Belgium) under the same environmental and edaphic conditions: substrate, watering regime, natural daylight, minimal temperature of 20 °C and relative humidity of the air. The developmental stages of coffee leaves are generally categorized as: (a) young leaves, (b) mature leaves, and (c) aged leaves. For this study, only the mature leaves (b) were collected. Mature leaves (b) are fully developed, and they do not show yet brown necrosis on the leaf margins [19]. The tree samples are conserved in Meise Botanic Garden and labeled with an identifier (*C. arabica* 2011027945, 19073828. *C. anthonyi* 2011033302, 2011033403, 20070347-77; *C. canephora* 19370050, 19800409) and herbarium vouchers are deposited in the Herbarium of the Meise Botanic Garden.

### 3.3. Sample Preparation

Leaf samples were dried after collecting by packing in sealed plastic bags filled with silica gel. The silica gel was replaced every day for one week. After one week, 1.0 g of the leaves was powdered for each sample with an ULTRA-TURRAX^®^ Tube Drive control (Q-Lab, Vilvoorde, Belgium). The powdering time was ten minutes at 6000 rpm for the leaves. Fruits (1.0 g) were ground for twenty minutes after a first quick grinding with mortar and pestle. The duration of grinding was defined to obtain a homogeneous powder. The extraction was performed by suspending 15.0 mg of powdered samples in 1.500 mL of Milli-Q water for 5 min in a 55 kHz ultrasonic bath [19]. Five sample extraction replicates were performed. Samples were filtered through a 0.2 µm cellulose acetate membrane and stored at −20 °C until analysis.

Fresh samples used for the phloem extraction were temporarily packed in sealed plastic bags and extracted by the phloem EDTA-facilitated exudation technique, as described by King and Zeevaart [35]. The concentration of the EDTA solutions was 20 mM in order to avoid cell damage and the optimal extraction time was 6 h, as previously tested for coffee samples [36]. The extraction was performed in total darkness and in a humid environment to avoid high transpiration which could result in a loss of exudates. The exudates were collected in 6.0 mL of EDTA solution and stored at −20 °C until analysis.

### 3.4. LC-HRMS Analysis

Semi-polar metabolite fingerprints were monitored using a 1200 series rapid resolution liquid chromatography (RRLC) system coupled to a 6520 series electrospray ionization (ESI)- quadrupole time-of-flight (QTOF) high-resolution mass spectrometer (HRMS) from Agilent Technologies (Waldbronn, Germany). A Poroshell 120 EC-C18 column (2.7 μm, 100 mm × 2.1 mm) with guard column (2.7 μm, 5 mm × 2.1 mm) from Agilent was used to carry out the chromatographic separation. The column temperature was set at 55.0 °C. The mobile phases were composed of 0.025% (*v/v*) of TFA (trifluoroacetic acid) and 0.075% (*v/v*) of FA (formic acid) in water (solvent A) and in acetonitrile (ACN, solvent B) for positive ion mode analysis. The same LC-MS conditions were applied to the negative ionization mode, but the additives of the solvent were changed. Solvent A was 20 mM ammonium formate pH 5.5 and solvent B ACN. The applied gradient was: 0 min, 0% B; 0–8 min, 0–10% B; 8–9 min, 10–12.5% B; 9–11 min, 12.5–15% B; 11–17 min, 15–80% B; 17–18 min, 80–100% B; 18–19 min, 100% B; 19–20 min, 100–0% B; post-run 8 min at 0.5 mL/min. The injection volume was 10µL. ESI-QTOF parameters were as follows: centroid, positive or negative mode, 2 GHz mode for resolution, mass range 100–1000 m/z, drying gas temperature and flow of 325 °C and 9 L/min respectively, nebulizer pressure 55 psi, and capillary voltage −4000 V. Nitrogen was used as the nebulizer gas. Data acquisition and LC-MS data analysis were carried out by MassHunter Acquisition^®^ software for QTOF (Version B.08), MassHunter Qualitative Analysis^®^ (Version B.10) software and MassHunter Quantitative Analysis^®^ (Version B.10) software (Agilent Technologies, Santa Clara, CA, USA). All samples were injected in random order and were analyzed in one batch. A quality control (QC) sample (a mix of all biological samples) was injected throughout the injection list after every 5 samples. An LC-autoMS/MS analysis was carried out to perform molecular networks. The conditions for the ESI-QTOF were set as follows: MS scan range 100–1700 m/z at 4 spectra/s; MS/MS scan range 50–1000 at 3 spectra/s; isolation width: medium mode (≈4 m/z); fixed collision energy was fixed at 25 eV. Max precursors at 3/cycle, threshold 500 absolute intensities. Precursor selection based on abundance dependent accumulation with scan speed at counts/spectrum with MS/MS accumulation time limit; active exclusion after 3 spectra released after 0.5 min and selection and sort of precursors by abundance for charges of 1 or 2.

### 3.5. Data Processing

ProteoWizard MSConvert tools (Version 3.03.9393, 64-bit) were used to convert raw data (.d Agilent ones) to .mzXML file. Peak Picking was chosen as a filter option. All data preprocessing and data processing were performed on the Workflow4Metabolomics infrastructure (https://workflow4metabolomics.org) (accessed on 15 April 2022). Detailed steps and parameters that were used for data processing are publicly available on the W4M workflow repository (https://usegalaxy.fr/u/amontis/h/coffee-published-2022) (accessed on 15 April 2022). Data preprocessing is based on XCMS software which allows performing the extraction of ions, their alignment across the samples, peak grouping, the retention time correction and annotation of isotope peaks, adducts and fragments. Matched Filter algorithms [37] were used with the parameters adapted for an Agilent 6520 series LC-QTOF. Filtering and normalization were then applied. Noise signal was estimated using blanks (injection solvent) as a reference. Signal drift correction was also performed (Batch correction tool). For each ion, an intra- and inter-batch correction were made by modeling the analytical effect using QC samples intensities according to the injection order. The regression model chosen for the batch correction step was a linear model. Variables with a coefficient of variation in the QC samples above 30% were then removed. Unsupervised multivariate analysis was carried out by performing a principal component analysis (PCA) which provided a prior overview of cluster separation. The parameters were set as follows: number of predictive components: 2; scaling: pareto; number of cross-validation segments: 4 to 7. The variables responsible for the separation between groups were initially provided by the loadings plots and then confirmed by Biosigner tool (with a seed value from 1 to 9). Biosigner is a tool including three different binary classifiers named PLS-DA (Partial Least Square Discriminant Analysis), RF (Random Forest) and SVM (Support Vector Machines), used to select the features which were found to be the most discriminant for each sample [20]. Student’s t-test and Kruskall–Wallis test with an applied *p*-value significance threshold ˂0.05 were performed to test each variable individually and to compare the probability distribution of the features observed on two or more samples.

The annotation step was firstly performed by carefully checking the information about the different m/z values in the available literature, then by computing the ion elemental formulas based on the m/z values with MassHunter Qualitative Analysis^®^ (Version B.10) from Agilent. Standard samples were also injected with the same LC conditions to confirm the identity of the most significant metabolites. In addition, data-dependent MS/MS acquisition was performed, and the acquired data were used to build a molecular network in order to have a global display of all related molecules. Data preprocessing for molecular networks were performed with MZmine 2 (version 2.53) (https://mzmine.github.io) (accessed on 20 April 2022) followed by MetGem 1.3.4 (https://metgem.github.io/) (accessed on 21 April 2022) software. The main databases used for the preliminary annotation step were PubChem and SciFinder^®^ online platforms. The main databases used for the annotation of the displayed molecules of molecular networks were GNPS library, PhytoChemical, Pesticides, NIH Natural Products, EMBL Metabolomics Core Facility, GNPS Collection Miscellaneous, GNPS Sigma’s Mass Spectrometry Metabolite and MIADP Spectral libraries. Preprocessing on MZmine 2 software was performed by setting the parameters as follows: range time of interest was set at 2–20 min with this part of the chromatograms being the richest in the focused compounds. Mass detection was performed by fixing the noise level at 2000 for MS1 and 50 for MS2. ADAP Chromatogram Builder was employed to build chromatograms by setting the parameters as follows: minimum group size of three scans, group intensity = 50 and minimum intensity = 50. The m/z tolerance was fixed at 0 or 50 ppm. Wavelet (ADAP) method was used for deconvolution by fixing a minimum feature height at 200 and a peak duration range at 0–5 min. The deconvolution was performed by fixing a value of 0.1 Da as m/z range for MS2 scan pairing and 0.5 min as RT range for MS2 scan pairing. Isotopic peak grouper was then performed with an m/z tolerance of 50 ppm, an RT tolerance of 0.2 min and a maximum charge of 2. Join aligner for data alignment was used to generate a peak list. The parameters were set as follows: m/z tolerance = 50 ppm, weight for m/z = 75, RT tolerance = 0.5 min, weight for RT = 25. Feature list filter was built by fixing m/z scan at 100–1000, RT range at 2–20, peak duration range at 0–5. Gap filled step was performed with 10% of intensity tolerance, m/z tolerance = 50 ppm and RT tolerance = 0.6. A filter to keep only peaks with MS2 scan (GNPS) was performed and data were then exported as .mgf files for spectra and .csv files as sample metadata files. Standard parameters were used to import Data “.mgf” and “.csv” in MetGem: m/z tolerance was set at 0.02 and minimum matched peaks were set at 4. A cosine score (CS, which represents the degree of spectra similarity between the spectrum of two molecules compared to each other) above 0.5 was applied to filter edges; maximum neighbor number (top K) of 10 and a max. connected component size of 1000 was set to build the networks.

### 3.6. Caffeine, Mangiferin and CQAs Concentration

Mass Hunter Quantitative Analysis software version B.10 (Agilent Technologies) was used to determine the concentration of caffeine, mangiferin and CQA isomers in the extracts. Caffeine concentration was determined by interpolation of caffeine calibration curve (y = −0.01141x^2^ + 606.5x + 826739.8; R^2^ = 0.9983) drawn from 0.001 to 200 μg/mL using a caffeine standard. Mangiferin concentration was determined by referring to a calibration curve (y = −0.001053x^2^ + 217.1x + 1014728.6; R^2^ = 0.9930) drawn from 0.01 to 100 μg/mL. CQAs concentration was determined by referring to a calibration curve (y = −0.000469257x^2^ + 127.9x + 9847.5; R^2^ = 0.9901) drawn from 0.001 to 300 μg/mL. The quantitation limit corresponded to the lowest concentration of each calibration curve. All these standard samples were also used to confirm the compound annotation through their m/z ratio and retention time.

## 4. Conclusions

Metabolomics studies allowed us to better investigate the chemical composition of the three analyzed coffee species and to improve our knowledge about *C. anthonyi*. If its fruits could be used as a substitute for *C. arabica* fruits for daily consumption, lower amounts of caffeine will be ingested—about two or three times less than the content of caffeine ingested with *C. arabica* fruits. Moreover, it should be considered that some important healthy molecules such as CQA derivatives could be introduced into the diet, being those compounds mainly found in *C. anthonyi* beans. However, xanthone and benzophenone derivatives, the main markers of *C. anthonyi* leaves and fruits, have not been detected in the beans, suggesting that these molecules are mainly synthesized and fully stocked in the fruit pericarp. The results suggested that if *C. anthonyi* could be employed as a component of new dietary supplements, both beans and pericarps should be used in order to completely exploit the healthy properties of this species. Meanwhile, further studies regarding the safety and organoleptic aspects are required. *C. anthonyi* leaves were also investigated and results highlighted the presence of large amounts of xanthones and CQA derivatives, while caffeine was almost absent. Naturally decaffeinated products can be made with coffee leaves from this species when the stimulating effects on the central nervous system due to this alkaloid need to be avoided. The investigation of the coffee phloem sap provided information that can be used to better understand the biochemical differences between species. Moreover, it was shown how a metabolomic fingerprint of the phloem sap is also effective to differentiate the genetically close coffee species. From this perspective, studying other coffee species by characterizing their extracts and by identifying the main markers could be helpful for a proper diet. Moreover, it would be interesting to study *C. anthonyi* in situ plants to see how the metabolome of this species can vary depending on the natural or in-field growing conditions when compared to the plants growing in the greenhouses. This has to be carried out in collaboration with the countries of origin.

## Figures and Tables

**Table 1:** Content of caffeine, CQA isomers and mangiferin (in mg/L) in *C. anthonyi*, *C. arabica* and *C. canephora* leaf extracts. Results are expressed as mg/L of extract (mean ± SD, *n* = 5), N.D. = not detected.

	*C. anthonyi*	*C. arabica*	*C. canephora*
Caffeine	N.D.	73 ± 3	51 ± 5
5-CQA	56 ± 3	19 ± 1	0.12 ± 0.01
4-CQA	6.4 ± 0.2	2.4 ± 0.4	N.D.
3-CQA	6.0 ± 0.5	4.6 ± 0.4	N.D.
Mangiferin	83 ± 4	4 ± 1	N.D.

**Table 2:** Content of caffeine, CQA isomers and mangiferin in the fruit extracts of *C. anthonyi* and *C. arabica*. Results are expressed as mg/L of extract (mean ± SD, *n* = 5) N.D. = not detected.

	*C. anthonyi*				*C. arabica*
	Entire Green Fruit	Entire Red Fruit	Bean	Pericarp	Entire Red Fruit
Caffeine	15 ± 2	8 ± 2	14 ± 2	0.05 ± 0.03	27 ± 4
5-CQA	11 ± 5	54 ± 7	109 ± 9	0.9 ± 0.2	0.2 ± 0.1
4-CQA	1.2 ± 0.2	17 ± 2	76 ± 5	0.8 ± 0.1	0.5 ± 0.1
3-CQA	1.0 ± 0.7	32 ± 2	87 ± 6	4.6 ± 0.4	N.D.
Mangiferin	11 ± 3	21 ± 4	N.D.	42 ± 7	N.D.

## Data Availability

Data are available at this link when you are logged on to the W4M platform: url: https://usegalaxy.fr/u/amontis/h/coffee-published-2022 (accessed on 15 April 2022).

## Supplementary Materials

The following supporting information can be downloaded at: https://www.mdpi.com/article/10.3390/molecules27103152/s1. Figure S1 shows loadings plots for leaves analyzed in ESI-MS (+) mode (A), the leaves in ESI-MS (−) mode (B), the phloem sap in ESI-MS (+) (C), and the fruits in ESI-MS (+) (D). Table S1 shows the list of the main annotated metabolites.

## Sample Availability

Extracts of samples are available from A.M. at Université Libre de Bruxelles. Biological samples are available at Meise Botanic Garden.
