# Supplementary material for: Targeted and Untargeted Mass Spectrometry-Based Metabolomics for Chemical Profiling of Three Coffee Species
Source: Molecules. 2022 May 14;27(10):3152. doi: 10.3390/molecules27103152 (PMC9143251; doi:10.3390/molecules27103152)
Supplement: Supplementary file 1 [file molecules-27-03152-s001.zip › molecules-1723639-supplementary.pdf]

Targeted and untargeted mass spectrometry-based metabolomics for chemical profiling of three coffee species

Andrea Montis [1,2,*], Florence Souard [3,4], Cédric Delporte [1,2], Piet Stoffelen [5], Caroline Stévigny [1], Pierre Van Antwerpen [1,2]

[1] RD3 Unit of Pharmacognosy, Bioanalysis and Drug Discovery, Faculty of Pharmacy, Université libre de Bruxelles, Campus Plaine, CP 205/09, 1050 Brussels, Belgium; cedric.delporte@ulb.be (C.D.); caroline.stevigny@ulb.be (C.S.); pierre.van.antwerpen@ulb.be (P.V.A.)

[2] APFP Analytical platform of the Faculty of Pharmacy, Faculty of Pharmacy, Université libre de Bruxelles, Campus Plaine, CP 205/5, 1050 Brussels, Belgium

[3] Département de Pharmacochimie Moléculaire, UMR 5063 CNRS, Université Grenoble Alpes, 470 rue de la chimie, 38400 Saint-Martin d'Hères, France; florence.souard@ulb.be (F.S.)

[4] DPP Department – Unit of Pharmacology, Pharmacotherapy and Pharmaceutical care, Faculty of Pharmacy, Université libre de Bruxelles, Campus Plaine, CP 205/5, 1050 Brussels, Belgium

[5] Meise Botanic Garden, Domein van Bouchout, Nieuwe laan 38, 1860 Meise, Belgium; piet.stoffelen@plantentuinmeise.be (P.S.)

* Correspondence: Andrea.Montis@ulb.be (A.M.)

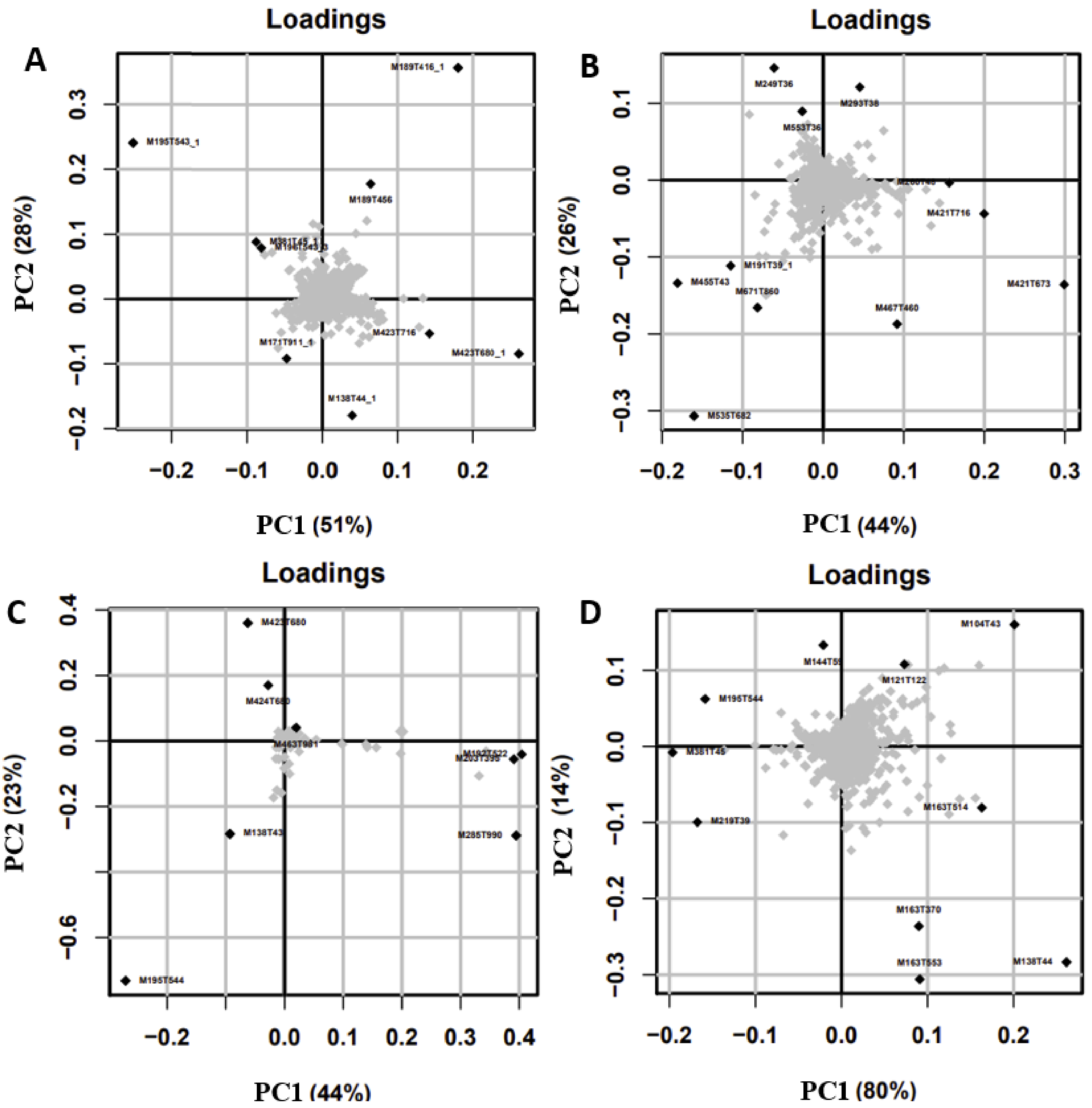

Figure S1. Loading plots showing the main metabolites responsible of the inter-species separation for the leaves analysed in ESI-MS (+) mode (**1A**), the leaves in ESI-MS (-) mode (**1B**), the phloem sap in ESI-MS (+) (**1C**), and the fruits in ESI-MS (+) (**1D**).

Table S1. List of the main annotated metabolites: caffeine, chlorogenic acids, benzophenone and xanthone derivatives.

RT (s)	m/z	Ion type	Name	Ion formula	Δppm
542	195.0874	$[M+H]^+$	caffeine	$C_8H_{11}N_4O_2$	-1.30
513	355.1025	$[M+H]^+$	5-CQA	$C_{16}H_{19}O_9$	0.40
578	353.0888	$[M-H]^-$		$C_{16}H_{17}O_9$	2.81
553	355.1020	$[M+H]^+$	4-CQA	$C_{16}H_{19}O_9$	-1.01
626	353.0887	$[M-H]^-$		$C_{16}H_{17}O_9$	2.53
370	355.1022	$[M+H]^+$	3-CQA	$C_{16}H_{19}O_9$	-0.45
461	353.0890	$[M-H]^-$		$C_{16}H_{17}O_9$	3.37
681	367.1044	$[M-H]^-$	5-FQA	$C_{17}H_{19}O_9$	2.57
759	367.1044	$[M-H]^-$	4-FQA	$C_{17}H_{19}O_9$	2.57
504	367.1038	$[M-H]^-$	3-FQA	$C_{17}H_{19}O_9$	0.94
513	337.0918	$[M+H]^+$	caffeoylshikimic acid	$C_{16}H_{17}O_8$	-0.01
560	517.1549	$[M+H]^+$	Glucopyranosyl-CQA	$C_{22}H_{29}O_{14}$	-0.43
680	423.0921	$[M+H]^+$	mangiferin	$C_{19}H_{19}O_{11}$	-0.21
673	421.0802	$[M-H]^-$		$C_{19}H_{17}O_{11}$	6.08
716	421.0799	$[M-H]^-$	isomangiferin	$C_{19}H_{17}O_{11}$	5.37
746	437.1078	$[M+H]^+$	homomangiferin	$C_{20}H_{21}O_{11}$	-0.09
738	435.0946	$[M-H]^-$		$C_{20}H_{19}O_{11}$	3.02
605	585.1450	$[M+H]^+$	neomangiferin	$C_{25}H_{29}O_{16}$	-0.02
589	583.1322	$[M-H]^-$		$C_{25}H_{27}O_{16}$	2.98
498	843.1609	$[M+H]^+$	tetrahydroxanthone-c-hexoside dimer	$C_{38}H_{35}O_{22}$	-0.65
491	841.1480	$[M-H]^-$		$C_{38}H_{33}O_{22}$	1.31
420	393.1182	$[M+H]^+$	garcimangosone D	$C_{19}H_{21}O_9$	-0.49
391	391.1048	$[M-H]^-$		$C_{19}H_{19}O_9$	3.43
551	571.1655	$[M+H]^+$	iriflophenone-di-O,C-hexoside	$C_{25}H_{31}O_{15}$	-0.43
537	569.1521	$[M-H]^-$		$C_{25}H_{29}O_{15}$	1.59
